# Video analysis of Achilles tendon ruptures in professional male football (soccer) reveals underlying injury patterns and provides strategies for injury prevention

**DOI:** 10.1007/s00167-023-07384-1

**Published:** 2023-03-28

**Authors:** Tim Hoenig, Thomas Gronwald, Karsten Hollander, Christian Klein, Karl-Heinz Frosch, Peter Ueblacker, Tim Rolvien

**Affiliations:** 1 Department of Trauma and Orthopaedic Surgery, University Medical Center Hamburg-Eppendorf, Martinistraße 52, 20251 Hamburg, Germany; 2 Institute of Interdisciplinary Exercise Science and Sports Medicine, MSH Medical School Hamburg, Hamburg, Germany; 3 Department of Sports Injury Prevention, VBG, German Statutory Accident Insurance for the Administrative Sector, Hamburg, Germany; 4 FC Bayern München Football Club, Munich, Germany; 5 Orthopaedics and Sports Medicine Practice, Munich, Germany

**Keywords:** Soccer, Football, Achilles tendon rupture, Biomechanics, Injury prevention

## Abstract

**Purpose:**

In professional football (soccer), Achilles tendon ruptures are severe injuries. Video analysis promotes a better understanding of the underlying situational and biomechanical patterns, and provides a roadmap for future research to improve the management and prevention of Achilles tendon ruptures. The purpose of this study was to identify injury patterns contributing to acute Achilles tendon ruptures in professional male football players.

**Methods:**

Professional male football players with an acute Achilles tendon rupture were identified using an online database. For every in-competition injury, the corresponding football match was detected. Video footage of the injury was accessed using Wyscout.com or publicly available video databases. Situational patterns and injury biomechanics of the injury frame were independently analysed by two reviewers using a standardised checklist and a motion analysis software. Finally, consensus was reached to describe the main injury patterns of Achilles tendon ruptures in professional male football players.

**Results:**

The search identified video footage of 80 Achilles tendon ruptures in 78 players. Most injuries (94%) occurred through indirect or non-contact mechanisms. The kinematic analysis revealed characteristic joint positions at the time of injury consisting of hip extension, knee extension, ankle dorsiflexion, foot abduction, and foot pronation in most cases. The underlying direction of movement was from flexion to extension (knee) and from plantarflexion to dorsiflexion (ankle). Player actions identified as main injury patterns were stepping back (26%), landing (20%), running/sprinting (18%), jumping (13%), and starting (10%).

**Conclusion:**

Most Achilles tendon ruptures in professional male football players are closed-chain indirect or non-contact injuries. Sudden loading to the plantarflexor musculotendinous unit remains to be the main component for most cases. By achieving a better understanding of underlying injury mechanisms, this study provides new strategies for the prevention of Achilles tendon ruptures.

**Level of evidence:**

Level IV.

## Introduction

The Achilles tendon is the strongest and thickest tendon in the human body [18]. Although it can withstand forces of up to 3800 Newton, Achilles tendon ruptures are common injuries among recreational and competitive athletes [21, 27, 28]. Regardless of the type of treatment [32], rehabilitation after an Achilles tendon rupture takes many months [9]. In fact, the average time to return to activity is six months as identified in a systematic review and meta-analysis [37].

Knowledge of situational and biomechanical patterns contributing to an Achilles tendon rupture is important for early (on-field) diagnosis, and for the development of prevention programmes [5, 7, 26]. To better understand the underlying mechanisms of Achilles tendon ruptures, a precise situational and biomechanical analysis of injury patterns appears crucial [7, 24]. However, despite numerous studies reporting on incidence, risk factors, diagnostic tools, and treatment [14, 20, 37], only few studies have addressed situational and biomechanical factors of Achilles tendon ruptures [1, 7, 11]. For this reason, several reports identified a gap of knowledge [1, 7, 11, 24].

Thus, the primary aim of this study was to identify situational and biomechanical factors contributing to acute Achilles tendon ruptures in professional male football players. It was hypothesised that a systematic video analysis can uncover characteristic injury patterns. Secondary aims were to report age and playing position of injured players, time to return to competition, and injury distribution across the season and match.

## Materials and methods

Ethical permission was not sought as only publicly accessible data were used. This approach is consistent with previous studies that have used the same methodology in other injuries [8, 15, 31].

### Injury identification

A database search was performed to detect acute Achilles tendon ruptures in professional football players. This methodology was recently used to successfully identify and evaluate injuries in professional soccer players [8, 15, 23, 33]. Injuries from the earliest record in 1995 until 2021 were identified from Transfermarkt.com [15]. In the present study, only players from the first and second national leagues were considered. For in-competition injuries, the corresponding match was detected using the above-mentioned online platform or media reports. Injuries that did not occur during competition were excluded. Video footage of each injury were accessed using Wyscout.com (Wyscout, Genova, Italy). In the case of missing video files, an additional web search was performed to search for video footage on other publicly available databases.

### Video analysis

Video analysis was performed to identify situational and biomechanical patterns. All analyses were performed independently by two reviewers (TH, medical doctor; TG, sports and exercise scientist). Both reviewers had experience in video analysis of sport injuries [13]. A standardised checklist (Table 1) was used for the evaluation of situational patterns. The checklist was based on a previously performed video analysis, with slight adaptations made to account for injury-specific biomechanical variables of the foot [17]. In short, situational patterns were described by field location, ball possession, direction of movement, and pitch contact, among others. Injury mechanisms were reported as contact, indirect contact or non-contact injuries [2]. Non-contact injuries were defined as injuries occurring without any contact to another player [2]. Indirect contact injuries were defined as contact to any body region except the lower leg or foot. Contact injuries comprised all injuries with another player’s contact to the lower leg and foot [2, 36]. A motion analysis software (Kinovea v.0.8.15, www.kinovea.org) was used for the evaluation of biomechanical patterns. In recent years, Kinovea has been widely used to assess sports injuries. Reliability and validity of this approach has been previously verified [34]. The kinematic analyses comprised trunk position (forward, neutral ± 10°, backward; earth vertical as reference) and lower extremity joint positions. In addition, the foot position in the coronal and axial plane at the assumed injury frame was evaluated.

**Table 1:** Predefined checklist for independent evaluation of situational and kinematic patterns. Modified from Klein et al. [17]

Situational pattern
Date of match
Match minute of injury / Match minute for injured player (if substitute)
Playing position of injured player
Field location (football pitch divided into 14 zones)
Ball possession
Injured player / Own team / Opponent team / Unclear / Nobody
Direction of movement
Forward / Lateral / Backward / Not horizontal
Pitch contact
Both legs / Single leg / No contact
Main injury mechanism
Contact / Indirect contact / Non-contact with distance to another player < 2 m / Non-contact with distance to another player > 2 m

Kinematics
Trunk position
Flexion / Neutral / Extension
Hip joint movement (sagittal)
Flexion to extension / None / Extension to flexion
Hip joint angle at assumed injury frame (sagittal)
< 45° / 45–90° / > 90°
Knee joint movement (sagittal)
Flexion to extension / None / Extension to flexion
Knee joint angle at assumed injury frame (sagittal)
< 45° / 45–90° / > 90°
Ankle joint movement (sagittal)
Plantarflexion to dorsiflexion/none/dorsiflexion to plantarflexion
Foot position in sagittal plane at assumed injury frame
Plantarflexion / Neutral / Dorsiflexion
Foot position in coronal plane at assumed injury frame
Supination / Neutral / Pronation
Foot position in axial plane at assumed injury frame
Abducted / Neutral / Adducted

**Table 2:** Characteristics of Achilles tendon ruptures in professional male football players. Situational and kinematic patterns are presented for each main injury pattern and the total cohort. RTC: Return to competition

Item	Jumping	Starting	Landing	Stepping back	Running/Sprinting	Other	All
*N*	10	8	16	21	14	11 Direct contact: 5 Twisting: 6	80
Player’s age at injury (years; mean ± SD)	31.2 ± 4.5	30.5 ± 5.9	29.1 ± 4.2	28.9 ± 4.5	26 ± 2.7	27 ± 3.3	28.6 ± 4.4
Time to return to competition (days; median [IQR])	219 [180–246] No RTC: 1	233 [187.5–227.5] No RTC: 1	202 [178.25–235.75]	184 [171.25–208.75]	242 [189,25–313]	197 [162–260]	202 [173–245] No RTC: 2
Minute of play (mean ± SD)	48.1 ± 27.8	54.6 ± 36.5	32.7 ± 20.2	37.3 ± 29.7	46.6 ± 31.6	36.7 ± 12.3	39.3 ± 26.7
Main injury mechanism (*n*): Direct contact / Indirect contact / Non-contact In brackets (n): Non-contact injuries with distance to another player < 2 meters and ≥ 2 meters	0/2/8 (6 + 2)	0/0/8 (3 + 5)	0/4/12 (7 + 5)	0/1/20 (10 + 10)	0/1/13 (7 + 6)	5/3/3 (3 + 0)	5/11/64 (37 + 27)
Player’s position (*n*): Goalkeeper / Defense / Midfield / Forward	0/6/3/1	2/1/5/0	2/7/7/0	1/6/10/4	0/7/6/1	1/3/4/3	6/30/35/9
Ball possession (*n*): Own team / Injured player / Opponent team / Unclear / Nobody	1/1/1/7	0/6/2/0	4/9/0/3	6/5/5/5	4/5/4/1	0/8/3/0	15/34/15/16
Direction of movement (*n*): No horizontal movement / Forward / Lateral / Backward	0/0/0/10	0/8/0/0	7/5/1/3	0/0/0/21	0/14/0/0	1/10/0/0	8/37/1/34
Pitch contact (*n*): Both legs / Single leg / No contact	7/3/0	4/4/0	1/15/0	16/5/0	0/14/0	0/10/1	28/51/1
Trunk position (*n*): Flexion / Neutral / Extension	3/7/0	7/1/0	5/11/0	14/6/0 Missing data: 1	NA	1/5/0 NA: 5	30/30/0 Missing data: 1 NA: 19
Hip joint movement sagittal (*n*): Flexion to extension / None / Extension to flexion	10/0/0	8/0/0	12/3/1	18/2/0 Missing data: 1	NA	2/3/1 NA: 5	50/8/2 Missing data: 1 NA: 19
Hip joint angle at assumed injury frame sagittal (*n*): < 45° / 45–90° / > 90°	10/0/0	7/1/0	14/1/1	20/0/0 Missing data: 1	NA	4/2/0 NA: 5	55/4/1 Missing data: 1 NA: 19
Knee joint movement sagittal (*n*): Flexion to extension / None / Extension to flexion	10/0/0	8/0/0	14/1/1	20/0/0 Missing data: 1	NA	3/1/2 NA: 5	55/2/3 Missing data: 1 NA: 19
Knee joint angle at assumed injury frame sagittal (*n*): < 45° / 45–90°/ > 90°	10/0/0	8/0/0	15/1/0	20/0/0 Missing data: 1	NA	5/1/0 NA: 5	58/2/0 Missing data: 1 NA: 19
Ankle joint movement sagittal (*n*): Plantarflexion to dorsiflexion / None / Dorsiflexion to plantarflexion	0/0/10	8/0/0	15/1/0	20/0/0 Missing data: 1	NA	4/2/0 NA: 5	47/3/10 Missing data: 1 NA: 19
Foot position in sagittal plane at assumed injury frame (*n*): Plantarflexion / Neutral / Dorsiflexion	3/6/1	0/0/8	0/1/15	0/0/20 Missing data: 1	NA	0/2/4 NA: 5	3/9/48 Missing data: 1 NA: 19
Foot position in coronal plane at assumed injury frame (*n*): Supination / Neutral / Pronation	0/6/4	0/5/3	0/9/7	0/13/7 Missing data: 1	NA	1/3/2 NA: 5	1/36/23 Missing data: 1 NA: 19
Foot position in axial plane at assumed injury frame (*n*): Abducted / Neutral / Adducted	6/4/0	4/4/0	9/7/0	10/10/0 Missing data: 1	NA	2/2/2 NA: 5	31/27/2 Missing data: 1 NA: 19

Disagreement was resolved by discussion and, if necessary, a third reviewer (KH). Kinematic analysis was only performed in case of sufficient video quality. Direct contact and running/sprinting-related injuries were excluded from kinematic analyses [13]. Finally, consensus was reached between the reviewers to determine and subsequently describe main injury patterns.

### Statistical analysis

For continuous variables, the mean (± SD) or median (interquartile range, IQR) was calculated. Other variables were described as absolute numbers or percentages of the total number of the corresponding observation. Microsoft Excel (Microsoft, Redmond, USA) was used for the analyses of this descriptive study.

## Results

### Identification of injuries

**Fig. 1:**
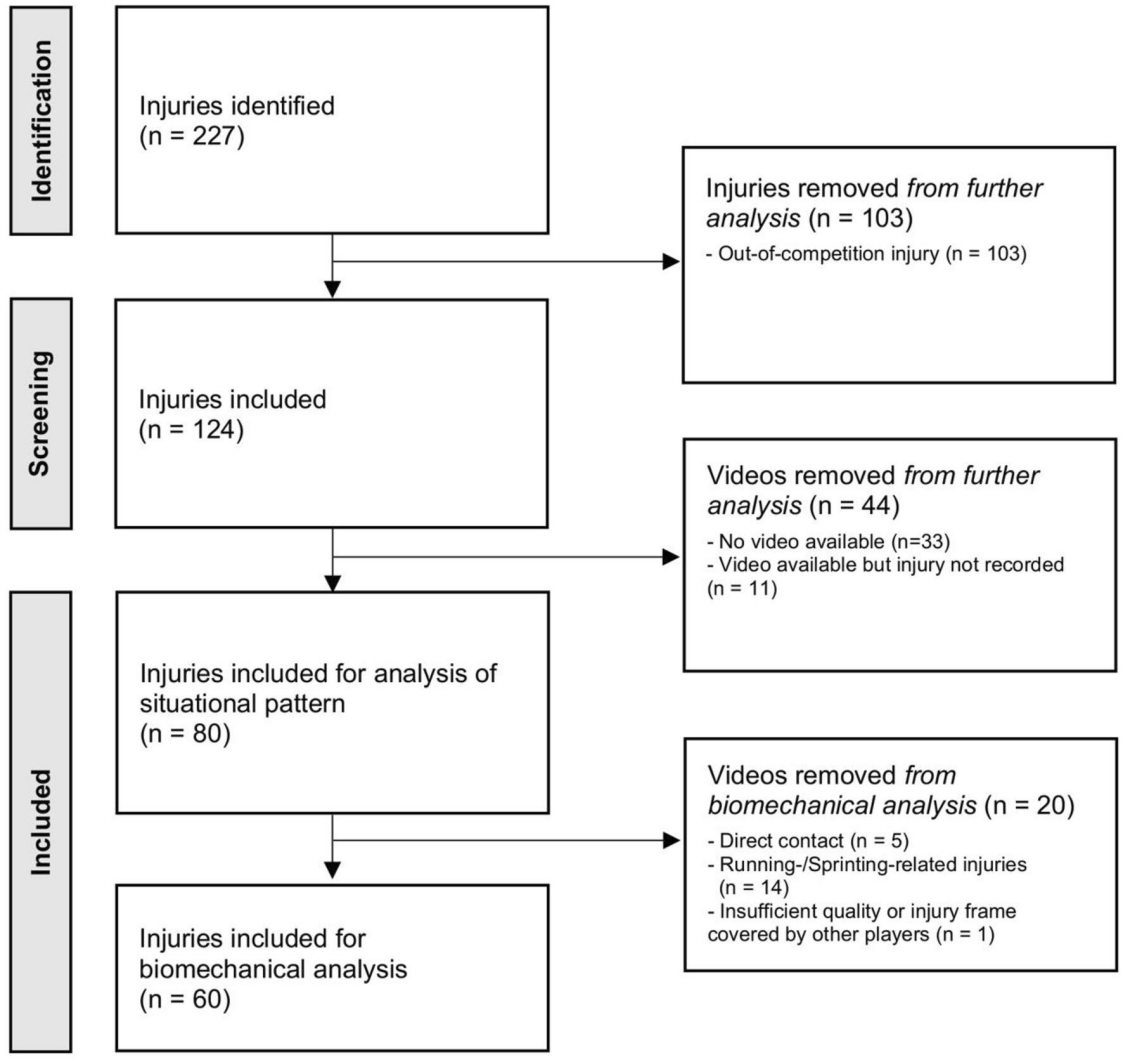
Flowchart of identification, screening and inclusion of cases

The initial search yielded 227 cases. Of these, 103 were out-of-competition injuries. Of the remaining injuries, 33 injuries were excluded due to missing video footage. Additionally, eleven injuries were excluded despite available video footage as video data of the actual injury frame was missing. Ultimately, 80 injuries from 78 players (two re-injuries) were included for video analysis (Fig. 1). Of these, 20 injuries were included for situational pattern analysis and 60 injuries for both situational and kinematic (biomechanical) analysis. Of all injuries with available video footage, one had five camera views, eight had four, 24 had three, 24 had two and 23 videos had one camera view. Videos had a frame rate of 25 frames per second and were of varying resolution, ranging from 960 × 544 pixel to 1280 × 720 pixel. The mean age of the players at the time of injury was 28.6 ± 4.4 years (Table 2). Return to competition at pre-injury level was confirmed in 78 players (rate of return to competition 98%). Median time to return to competition was 202 days (IQR 173–245).

### Situational patterns

**Fig. 2:**
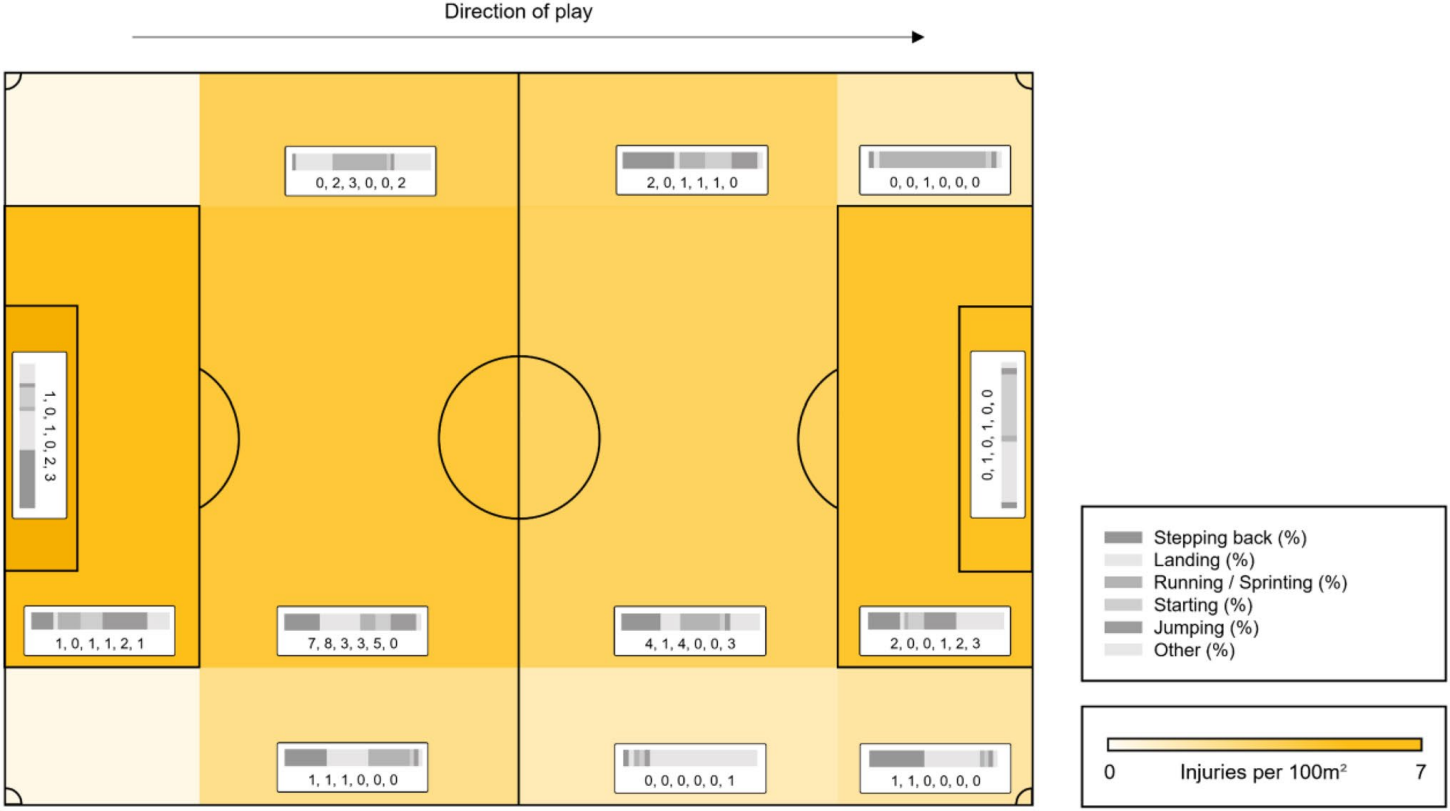
Distribution of Achilles tendon ruptures by field location. A football pitch was divided into 14 zones with yellow colouring indicating higher injury prevalence. Likewise, the distribution of main injury patterns is presented for every zone by both percentage value (grey bar graph) and absolute count (numbers below)

**Fig. 3:**
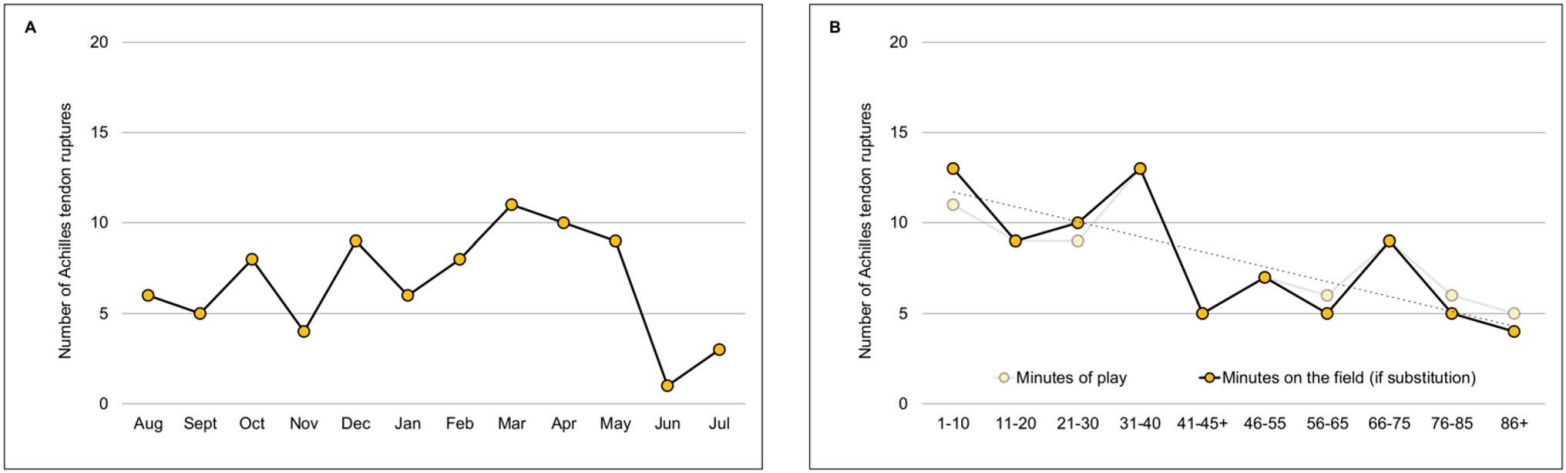
Distribution of Achilles tendon ruptures throughout the season (**A**) and match (**B**)

Injuries were classified as being direct in five cases, indirect in eleven cases, and non-contact in 64 cases, respectively. Achilles tendon ruptures tended to occur more frequently within the team’s own half (49 injuries in the team’s own half compared to 31 injuries) as visualised in Fig. 2. Injury occurrence throughout the season and match is demonstrated in Fig. 3.

### Underlying biomechanical factors

Of the 60 cases in which a kinematic analysis was performed, the trunk was in a forward (50%) or neutral (50%) position (Table 2). The hip joint moved from flexion to extension in most cases (83%) with the hip being extended (i.e., < 45° flexion) at the time of injury (92%). The knee joint moved from flexion to extension in 55 cases (92%) with the knee being in extension (i.e., < 45°) in most cases (97%). The ankle was performing a dorsiflexion movement in 47 cases (78%). In the coronal and axial plane, the foot was mostly pronated/neutral and abducted/neutral (98 and 97%, respectively).

### Main injury patterns

The injury patterns identified as main patterns were stepping back (*n* = 21; 26%), landing (*n* = 16; 20%), running/sprinting (*n* = 14; 18%), jumping (*n* = 10; 13%) and starting (*n* = 8; 10%). Other injury patterns were found in eleven cases (14%), namely six twisting injuries and five direct contact injuries. Detailed underlying situational patterns and kinematics for each main pattern are presented in Table 2.

## Discussion

The most important finding of the present study was that video analysis of 80 Achilles tendon ruptures in professional male football players revealed typical underlying situational and biomechanical patterns. The identified main injury patterns of acute Achilles tendon rupture were *stepping back, landing, running/sprinting, jumping* and *starting*. All injury patterns relied on a high and sudden loading to the plantarflexor musculotendinous unit. Most injuries (94%) were indirect or non-contact injuries. All main patterns were closed-chain movements (fixation of the affected leg to the ground). The kinematic analysis of the assumed injury frame revealed a change from knee flexion to knee extension. The ankle movement at the assumed injury frame was from plantarflexion to dorsiflexion in most cases. Typical joint positions at the assumed injury frame were hip extension, knee extension, and ankle dorsiflexion. Foot abduction and pronation was often seen but neutral positing of the foot in the axial and coronal plane may also be present.

**Fig. 4:**
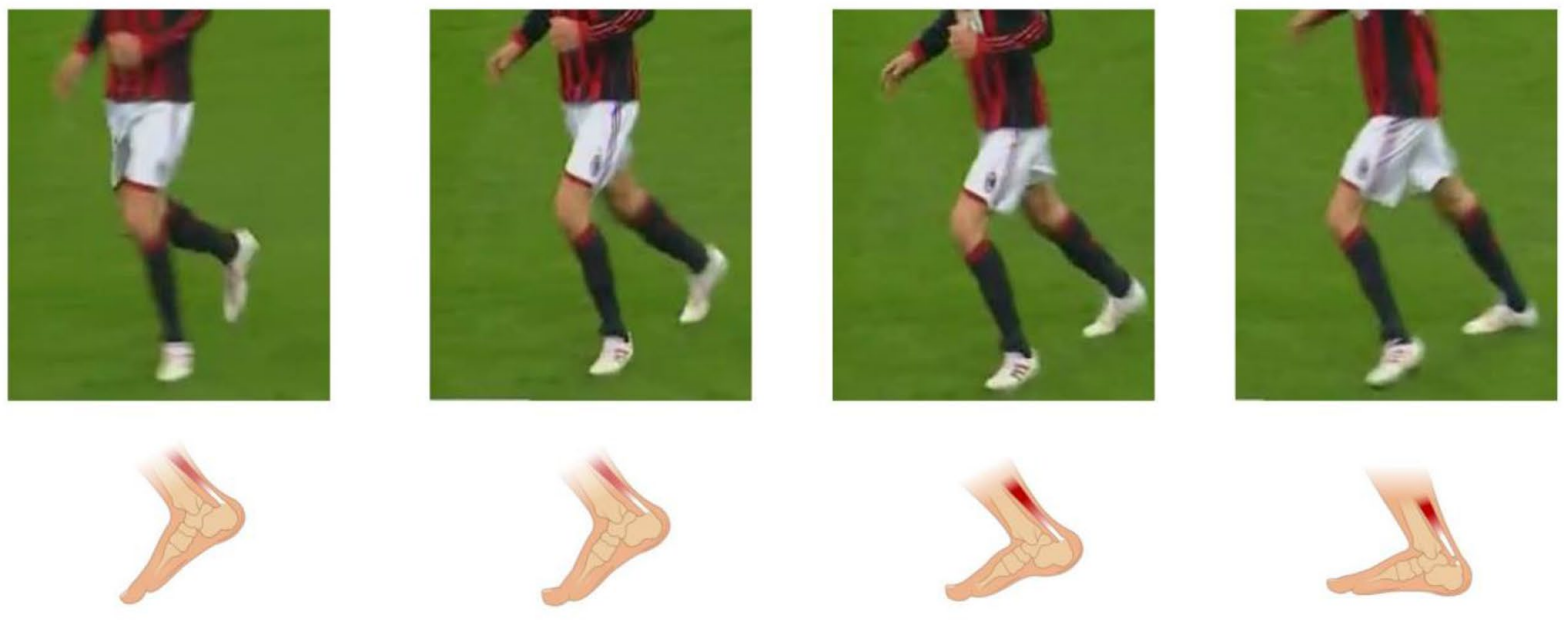
Biomechanical model of a “stepping back” Achilles tendon rupture. Performing a “stepping back” movement was the most frequent injury pattern leading to an Achilles tendon rupture in professional male football players. Initial direction of movement is backward with the affected leg being used to initiate change of direction. This movement applies a high force to the Achilles tendon over a short period of time. The Achilles tendon injury is elongated at the assumed injury frame due to ankle dorsiflexion and a knee extension movement. The hip is in extension with the trunk being in a forward position. Foot abduction and pronation is commonly seen but neutral positioning of the foot in the axial and coronal plane may also be present. Video footage (Serie A, 14.03.2010, Sky/Wyscout/YouTube) is publicly available and protected by European copyright laws for scientific use

*Stepping back* was found to be the most common injury pattern (Fig. 4). It is characterised by a sudden dorsiflexion of the plantarflexed foot. This movement is commonly performed to stop a backward movement and to initiate change of direction (in order to aim for subsequent forward acceleration). Consequently, this injury pattern is related to and may overlap with a *starting* injury pattern. Likewise, *jumping* and *starting* are closely related injury patterns with the player pushing-off horizontally or vertically. Of note, all jumping injuries occurred during backward running (player running backward, then performing a jumping movement). The fourth pattern is *landing* which also includes sudden dorsiflexion of the plantarflexed foot. Similar to a previous report [13], the assumed injury frame in *running/sprinting* is difficult to detect on video recordings. However, it was previously assumed that an Achilles tendon rupture occurs in the mid or late stance phase due to its high demand on eccentric contraction [7]. However, the concept of eccentric contraction theory of tendon ruptures was challenged recently. In fact, the triceps surae muscle may possibly load the Achilles tendon concentrically from proximal during the stance phase [4, 19]. Together with a forcefully performed ankle dorsiflexion from distal, loading on the Achilles tendon may exceed the tendon loading capacity. Of note, elongation of the Achilles tendon can be seen with both ankle dorsiflexion and knee extension due to the multiarticular anatomy of the gastrocnemius muscle.

All main injury patterns rely on similar kinematics with the hip being in extension and the knee being in late extension (direction of movement: flexion to extension). Except for the jumping pattern, the ankle was in a dorsiflexed or neutral (direction of movement: plantarflexion to dorsiflexion) position at the assumed injury frame. While these injury patterns accounted for most injuries, six injuries occurred during a twisting movement with varying kinematics. For this reason, no main injury pattern was described in this study for twisting injuries, but clinicians should acknowledge this injury mechanism. Five injuries occurred due to direct contact to the affected leg or foot which mainly also resulted in a sudden twisting movement of the lower leg and foot.

As early as in 1959, Arner & Lindholm [1] stated that musculotendinous units are at highest risk to rupture if passive stretching occurs in a contracted muscle. Since then, aetiology and pathophysiology of Achilles tendon ruptures have been widely discussed [14, 24]. Risk factors contributing to Achilles tendon ruptures include age, male sex, previous injuries such as tendinopathy, systemic diseases, footwear, genetics, or medications [24]. However, biomechanical issues were rarely addressed as only few studies investigated Achilles tendon rupture mechanisms. Back in 1882, Maydl [29] postulated that Achilles tendon ruptures typically occur during (1) landing on the heel/toe, (2) forefoot push-off (e.g. in dancers), (3) standing up on the toe, or (4) rising up from a chair. In 1959, Arner & Lindholm [1] interviewed 92 patients following Achilles tendon ruptures to elicit injury mechanisms. In their case series, the most common injury type was a forefoot “push-off” and simultaneous knee extension movement (e.g. sprint start or running movement). The second most common type was an unexpected dorsiflexion of the foot in neutral position (e.g. tripping into a hole). The third type was a sudden dorsiflexion movement of the plantarflexed foot (e.g. landing on the forefoot). Only one out of 92 ruptures was thought to be due to direct trauma. Although this report was already published in 1959 without any access to video recordings, a recent video analysis of Achilles tendon ruptures in basketball showed similar results with push-off movements being the most common injury pattern [22]. In 2019, a case report by De la Fuente et al. [7] evaluated the kinematics of an Achilles tendon rupture that was captured on video. Again, this video evaluation analysed a “push-off” Achilles tendon rupture in a track and field athlete.

Video analysis of sports injuries is of major clinical relevance which may explain its high popularity in recent years [8, 13, 16, 17, 25, 35, 36]. Many articles have been published describing mechanisms of injury to the anterior cruciate ligament [8, 25, 36], adductor longus muscle [35], and hamstring muscles [13], among others. However, despite a high injury severity in professional male football, evidence was lacking for Achilles tendon ruptures [15]. In basketball, eight out of twelve Achilles tendon ruptures (67%) were characterised as “running takeoff” injuries [22]. This finding is not generally contrary to our finding as “starting” was identified to be a main injury pattern in professional male football. However, the authors concluded that there is little support to claim that jumping is a common injury mechanism. This may indeed be true for professional basketball players, and excellent jumping skills may be seen as a prerequisite for a successful basketball career. Nevertheless, this assumption does not apply for male professional football as 26 Achilles tendon ruptures were associated with a jumping movement. In addition to the previous literature findings, we identified and clarified further injury mechanisms of Achilles tendon ruptures, e.g. stepping back. The divergence may be explained by the specific demands of the respective sports and (maybe) the much larger cohort within this report. With regards to secondary aims, findings on mean age at injury, distribution of injury throughout the match, distribution of injury throughout a football season and time to return to competition were comparable to previous reports [10, 12].

A profound insight into injury aetiology may support translation towards future development of prevention programmes [3, 6]. By identifying situational and biomechanical patterns of injuries, specific training programmes for the prevention of Achilles tendon ruptures may be designed. These programmes can be implemented for either a whole football team or athletes at risk. For instance, it is well-known that athletes with chronic Achilles tendinopathy or a prior Achilles tendon rupture are at elevated risk and may particularly benefit from preventive measures [24]. With increasing knowledge of the underlying biomechanics of acute musculotendinous injuries, it may be stated that the underlying aetiology should already be addressed during training, thus being not only the “problem” but also the “solution” [13]. Therefore, findings from this study may possibly serve as a reference for supportive high-energy loading exercises in prevention programmes. However, current recommendations to prevent Achilles tendon ruptures are not based on strong evidence [14]. A considerable number of studies has been published on Achilles tendinopathy and, potentially, similar recommendations may apply [14, 30]. Nevertheless, the efficacy of preventive measures remains to be studied [14, 30]. Findings from this study may also be used for further research on treatment and rehabilitation of Achilles tendon ruptures. At every stage of managing Achilles tendon ruptures, e.g. initial surgical treatment, clinicians should acknowledge that there are situations and kinematics in which the Achilles tendon is most vulnerable and prone to rupture.

The main strength of this study is its systematic approach with the identification of 80 injuries and a standardised assessment by independent reviewers. In contrast, it is an important limitation that the Achilles tendon rupture diagnosis was based on non-scientific reports (mostly press releases). However, it seems generally unlikely that the diagnosis of an Achilles tendon rupture was incorrectly stated to the media given its severity and long time to return to competition [12, 33].

## Conclusion

Most Achilles tendon ruptures in professional male football are closed-chain indirect or non-contact injuries. Sudden loading to the plantarflexor musculotendinous unit remains to be the main component for most cases. Typical inciting events are stepping back, landing, running/sprinting, jumping and starting. Other than these patterns are present but rare. Typical underlying kinematics are: trunk in forward position, hip in extension, knee joint in late extension (movement: flexion to extension), ankle in dorsiflexion (movement: plantarflexion to dorsiflexion), and foot in abduction and pronation. Findings form this study can be used for the development of prevention programmes. Future video-based analyses of Achilles tendon ruptures are encouraged to further specify the similarities and differences between sports.

## Data Availability

Further data are available upon request.
